# Connecting the Dots: Finding Continuity Across Visuospatial Tasks and Development

**DOI:** 10.3389/fpsyg.2019.01685

**Published:** 2019-08-02

**Authors:** Sammy Perone, Vanessa R. Simmering

**Affiliations:** ^1^Department of Human Development, Washington State University, Pullman, WA, United States; ^2^Department of Psychology, McPherson Eye Research Institute, and Waisman Center, University of Wisconsin-Madison, Madison, WI, United States; ^3^ACTNext by ACT, Inc., Iowa City, IA, United States

**Keywords:** dynamic field theory, dynamic neural field model, developmental process, domain general cognitive processes, visuospatial cognitive development

## Abstract

The study of cognition and its development has long been partitioned into sub-domains, with different tasks designed to assess different constructs and for use during different developmental periods. A central challenge is to understand how a single cognitive system organizes itself across many contexts and developmental periods in which we study it. This article takes a step toward tackling this challenge through a theoretical review of simulations of a dynamic neural field (DNF) model of visuospatial cognitive development. The DNF model simulates basic neurocognitive processes of encoding, maintenance, and long-term memory formation that are coupled to different behavioral systems to generate behaviors required across different tasks used with different age groups. The model simulations reviewed here were initially focused on explaining performance in specific experimental conditions within a developmental period. This article brings to the forefront the larger theoretical goal to understand how a set of basic neurocognitive processes can underlie performance in a wide array of contexts. This review connects behavioral signatures and developmental phenomena from spatial cognition, infant visual exploration, and capacity limits in visual working memory into a single theoretical account of the development of basic visuospatial cognitive processes. Our synthesis yielded three new insights not evident when considering the model simulations in isolation. First, we identified behavior as an emergent product of the neurocognitive processes at work in the model, task context, and development. Second, we show the role of stability of perceptual and memory representations to support behavior within a task and across development. Third, we highlight continuity of ongoing real-time processes at work within and across tasks and over development.

A fundamental question driving psychological science is, how is behavior connected across the many contexts in which we study it? To make the study of cognition and development tractable, researchers have partitioned the field into sub-domains, with different tasks designed to assess different constructs and for use with different age groups due to behavioral limitations in infants or young children. This has led to minitheories of performance that are closely tied to the task, domain, and developmental period for which they were developed. How such minitheories should be connected has sparked debate over whether there is continuity or discontinuity over development and whether behavior reflects domain general or domain specific processes (e.g., Kagan, 2008). The isolation of cognitive processes was needed to understand the complex processes under study, and has provided a broad empirical and theoretical foundation on which we can begin to “put the pieces together again” (Oakes, 2009). Here, we aim to put together pieces from historically distinct domains by synthesizing a set of model simulations of visuospatial cognitive development. The model simulations initially focused on explaining behavior in specific task contexts and developmental periods. Our synthesis enabled us to identify a common set of principles that enable a single cognitive system to perform a variety of visuospatial tasks and the central developmental mechanisms underlying behavioral change.

The simulations we review were conducted using a Dynamic Neural Field (DNF) model developed in the Dynamic Field Theory (DFT), a theory of embodied cognitive dynamics that is rooted in dynamic systems theory (Schöner et al., 2015). DFT construes behavior as an emergent product of multiple forces, including the developmental state of the child, task context, out-of-lab experience, and behavioral history in a task (see Thelen et al., 2001; Spencer et al., 2008; Perone and Simmering, 2017). The goal of DFT is to explain the relation between brain and behavioral dynamics in real time and over development. DFT assumes a set of general principles govern behavior across a wide array of tasks. Our synthesis illustrates how a single system can organize itself within a specific task context over development. Relative to other modeling approaches in cognitive development, DFT emphasizes behavior and real-time processes with less attention to long-term learning such as concept formation (see Schlesinger and McMurray, 2012, for comparison of models in cognitive development). Many core DFT features are shared with non-computational process-based explanations of behavior (e.g., Smith and Thelen, 2003; for discussion, see Perone and Simmering, 2017).

The simulations we review fall into three general domains: spatial cognition, infant visual exploration, and visual working memory. Our review is organized in the approximate chronological order the work was initiated to provide an account of the theoretical insights gained by connecting work across these domains, as well as how we initially made those connections (see also Spencer et al., 2009; Ambrose et al., 2015). We make brief reference to the competing theories in each domain, but do not discuss theoretical contrasts in depth; readers interested in such comparisons, as well as nuanced model simulation details, can find them in the original papers.

## Theoretical Constructs

Three theoretical constructs were identified by bringing the simulations together here. The first construct is *emergence*, which refers to phenomena that arise from the interaction of multiple elements, with none of the elements individually defining the outcome. These simulations show patterns of behavior emerge in a specific task and developmental context: behavior results not just from cognition but from the interaction of cognition with specific task contexts through the body (Thelen and Smith, 1994). The second construct is *stability*, which is the driving force of developmental change in children’s task-specific performance. Stability refers to how reliably a system enters a given state. Stability plays out in real time (e.g., generating a motor plan to reach for an object) as well as over development (e.g., efficiently reaching to the desired location). The simulations reviewed address the sources of developmental change in stability within the cognitive system as well as the consequences of increasing stability for children’s performance across tasks and domains. The third construct is *continuity* of processes over time and across contexts. Continuity over time bridges timescales, from real time to learning and development. In real time, the same ongoing neural processes in the model implement the cognitive processes of encoding, maintenance, comparison, recognition, and novelty detection; they are emergently distinct, arising through the structure of the task, not a change in how model operates. Through learning and development, the same real-time processes underlie task performance but may become more advanced as they build on the history of the system, increasing in stability and using new knowledge as it is acquired. Across contexts, our simulations show continuity with the same underlying neurocognitive processes producing a wide range of behavioral phenomena when situated in different tasks spanning domains and developmental periods.

## Overview

The review is organized as follows. First, we describe the DNF model and neurocognitive processes it simulates. Next, we describe the neurodevelopmental mechanism responsible for neurocognitive and behavioral change – called the Spatial Precision Hypothesis – in the simulations we review. After that, we synthesize simulations in the domains of spatial cognition, infant visual exploration, and visual working memory capacity. We close by offering concluding remarks about contributions of the model simulations to long-standing debates in cognitive development about the nature of developmental change in cognition.

## Dynamic Neural Field Model

Box 1 shows the DNF model and provides a brief summary of the dynamics of its behavioral and neurocognitive systems. The neurocognitive system consists of a three-layer architecture: two excitatory layers, referred to as the contrast and working memory layers, which interact through a shared layer of inhibitory neurons (not shown for simplicity). The contrast layer serves to encode items into the working memory layer and to detect novelty. It is referred to as a contrast layer because a key function of the layer is to detect differences between what is in working memory and new items in the environment. The working memory layer maintains items in the absence of input, that is, after items are no longer present in the task space. The contrast and working memory layers are coupled to memory trace layers that facilitate encoding and working memory formation through Hebbian learning. We will show how the continuous interactions among these layers, shown with green (excitatory) and red (inhibitory) arrows in Box 1, implement the cognitive processes necessary for a wide range of behavioral tasks: encoding, maintenance, recall, comparison, recognition, and novelty detection.

BOX 1 **Overview of components of Dynamic Neural Field model and behavioral systems.**

**Dynamic Neural Field Model**. The Dynamic Neural Field (DNF) model consists of a three-layer neurocognitive system coupled to fixation and same-different decision systems. The model encodes stimuli in the task space into working memory and forms long-term memories. The coupling of behavioral systems to the neurocognitive system can produce behavioral signatures of novelty detection, recognition, and recall across a wide array of tasks and periods of development.

**Fixation System**. The model can be equipped with a fixation system that looks at task-relevant locations at which stimuli appear, including left, right, and center locations. When the fixation system looks at a location, the stimulus at that location (e.g., red star in the center) is input into the contrast layer of the neurocognitive system where it is encoded into the working memory layer. The fixation system can also look at away locations at which no stimuli appear.

**Decision System**. The model can be equipped with a same-different decision system. This system responds “same” when an item is maintained in the working memory layer that matches an item in the task space, and it responds “different” when an item is encoded in the contrast layer that is not actively being held in the working memory layer.

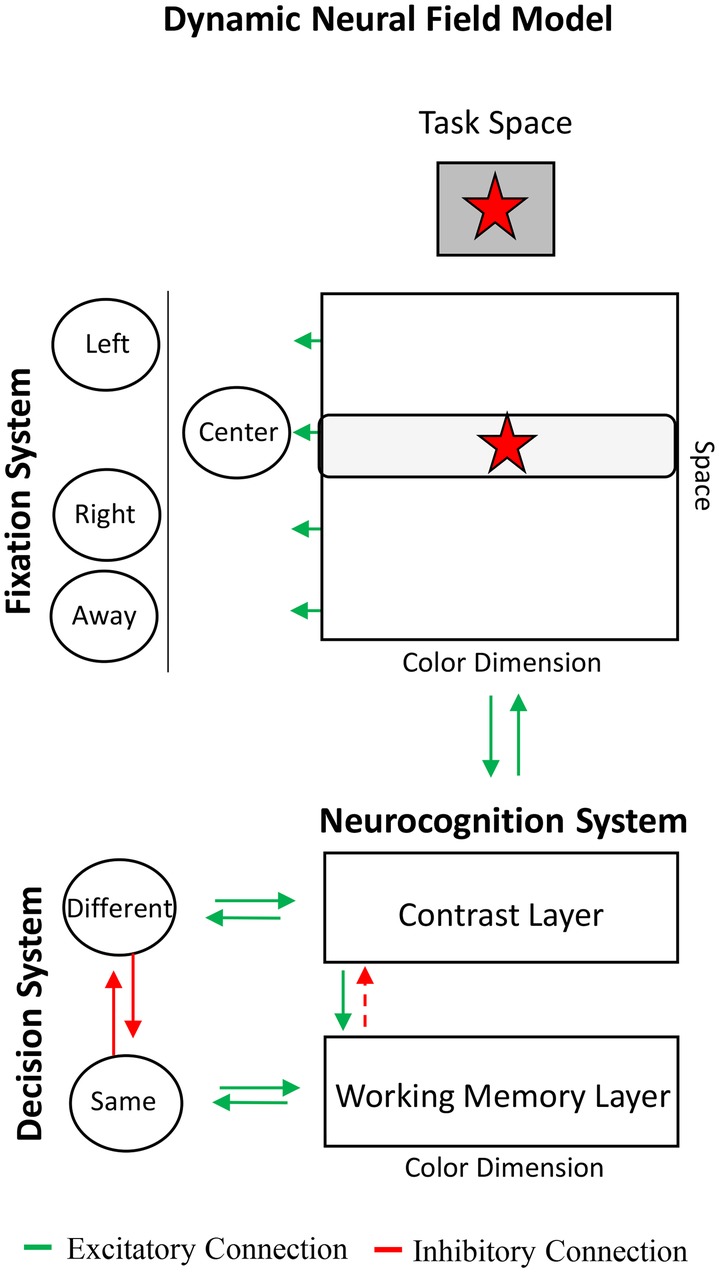

**Neurocognitive System**. The neurocognitive system encodes and forms working memory representations for stimuli over continuous dimensions (color, location). The architecture is built from a contrast layer that takes input from the task space (green arrow from task space to contrast layer). The contrast layer encodes stimuli from the task space into a working memory layer (green arrow from contrast to working memory layer). These layers are coupled to memory trace layers (not shown), which facilitates encoding and working memory formation for previously experienced stimuli using a form of Hebbian learning. These layers also interact through a shared layer of inhibitory neurons (not shown for simplicity). The dashed red arrow from the working memory to the contrast layer highlights the functional connection in which strong activity in working memory suppresses activity associated with the stimulus in the contrast layer.

Layers in the DNF model are neural fields organized topographically such that neighboring neurons within a field represent similar values along a dimension (e.g., shades of blue). In a neural field, a stimulus excites selectively-tuned neurons (i.e., the presence of a blue input excites neurons that respond to blue). Interactions among local excitatory and lateral inhibitory connections (termed the “interaction profile”) lead a “peak” of activation to emerge. The peak is a self-organized pattern of neuronal activation that estimates the stimulus. When the input is removed and local excitatory connections are weak, the peak decays. This is the encoding state in which neurons are only active when an input is present. When the input is removed and local excitatory connections are strong, the peak can be maintained in the absence of input. Peaks leave memory traces that facilitate the re-formation of peaks that estimate similar features at future points in time.

**Figure 1:**
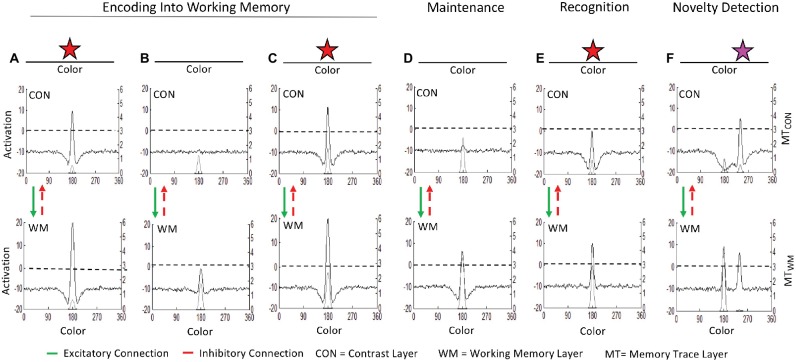
The ongoing neurocognitive processes in the three-layer dynamic neural field (DNF) model: **(A)** Initial presentation of a stimulus (red star) generates a suprathreshold (>0) peak at sites tuned to its hue in the contrast layer (CON). CON passes excitatory input to associated sites in the working memory (WM) layer. Activity in both layers leave memory traces (MT; gray line, right y-axis). **(B)** When the stimulus is removed, activation subsides in both CON and WM. **(C)** When the same stimulus is presented again it continues to be encoded into WM. **(D)** When the stimulus is removed again the accumulated memory trace enables WM to maintain an activation peak despite the absence of input. **(E)** When the same stimulus is re-introduced again, the peak in WM inhibits similarly tuned sites in CON through the shared inhibitory layer (not shown). Activation in CON remains subthreshold, which is the mechanism of visual recognition in the model. **(F)** When a new stimulus (magenta star) is presented, activation in CON rises to suprathreshold levels, signaling novelty detection.

Figure 1 illustrates how the three-layer neurocognitive system in Box 1 simulates the basic cognitive processes at work in a simple visual task with stimuli represented along a color dimension. Description of the behavioral systems will be introduced when describing specific simulations requiring each system. Figures 1A–D show the encoding of a stimulus into working memory. Figure 1A shows the model’s initial response when a red star is input to the contrast layer. This leads to a suprathreshold (>0) peak in the contrast layer at neurons selectively tuned to its hue which, in turn, generates a peak in the working memory layer (black lines, left y-axis). These peaks leave excitatory traces in corresponding memory trace fields (light gray line originating at zero on the right y-axis) which strengthen the connectivity among active neurons in a Hebbian fashion, facilitating the formation of peaks for similar stimuli in the future. Figure 1B shows the state of the model when the stimulus has been removed. Activity in the contrast and working memory layers decay. When the stimulus is presented again in Figure 1C, the neuronal response in the contrast and working memory layers is strong due to the acquired memory traces. When the stimulus is removed once again in Figure 1D, the working memory layer maintains a peak associated with the red stimulus despite its absence.

Now that the model has encoded an item into the working memory layer, the same neurocognitive processes that encoded and maintained the item support comparison of new inputs to the contents of the working memory layer. Figures 1E,F show the comparison process and how it leads to recognition of familiarity and detection of novelty, respectively. When the familiar red star is presented in Figure 1E, the robust peak in working memory inhibits associated sites in the contrast layer *via* the shared inhibitory layer (not shown for simplicity). Consequently, activation at the associated site in the contrast layer (i.e., the same color value held in memory) is suppressed. This is the mechanism of recognition in the model: the presence of a peak in the working memory layer inhibits further encoding of similar items (i.e., subthreshold activation in the contrast layer in Figure 1E). Because activation in the contrast layer does not surpass threshold, no further activation projects to the working memory layer, leading to a smaller peak relative to when the stimulus was being encoded (c.f., working memory layers in Figures 1A,C). When a novel stimulus is presented, such as the magenta star in 1F, a peak in the contrast layer emerges. This is the mechanism of novelty detection in the model: with no similar item (peak) in the working memory layer, a new item may be encoded in this region of the contrast and working memory layers (i.e., suprathreshold activation in the contrast layer in Figure 1F). Thus, the processes that support encoding can also serve as signals of familiarity and novelty relative to what information is already held in memory.

The comparison process shown in Figure 1 illustrates accurate recognition of familiarity and detection of novelty, but this is not necessarily the case. As these processes operate in real time in the model, comparison is influenced by distortions and errors in memory. For example, the model may “forget” an item if the peak in the working memory layer spontaneously decays from neuronal noise, a weak memory trace, and/or parameter settings with weak local excitation (described further below). A peak can also be “knocked out” by the formation of other peaks nearby (i.e., for similar items) if the incoming peak generates lateral inhibition that suppresses activation of the nearby peaks to subthreshold levels (Johnson et al., 2014). Distortions in the working memory layer may occur when sources of excitation or inhibition cause the peak to localize at an inaccurate feature value, for instance due to the influence of perceptual reference frames (described in the spatial recall and position discrimination sections below), due to long-term memory traces (described in the spatial recall section below), or due to additional inputs (e.g., Johnson and Spencer, 2016; Schutte and DeGirolamo, 2019).

Notably, the same continuous processes encode, maintain, and compare items in the task space to those in memory. When new stimuli are presented and the working memory layer contains no similar items, the item is encoded. If a similar item is already held in the working memory layer, the new item is compared. When input is removed, the same local excitatory and lateral inhibitory processes that encoded the item in the contrast layer maintain the item in the working memory layer. In the DNF model, distinctions between cognitive processes emerge as the neurocognitive system interacts with the world (e.g., encoding becomes novelty detection, maintenance in working memory becomes recognition). Pezzulo and Cisek (2016) proposed the goal of brain is to interact with the world rather than represent it. The DNF model is situated within this realm. Our review will highlight how behavior, perceptual processes, and cognitive or memory processes interact in specific task contexts.

### Spatial Precision Hypothesis

The developmental mechanism responsible for all the behavioral changes we describe here was guided by the Spatial Precision Hypothesis, which posits excitatory and inhibitory interactions grow stronger with age (see Simmering and Schutte, 2015, for review). Implementing the Spatial Precision Hypothesis involves strengthening the degree to which the excitatory and inhibitory layers interact. This strengthening is normally achieved by hand-tuning the parameters that govern these interactions. When the excitatory and inhibitory connections are strengthened, stability of the system increases. Increased stability in real time has five consequences for performance across the tasks we review here (see Simmering, 2016, for additional consequences): (1) activation builds more quickly in the contrast layer for faster encoding; stronger activation produces peaks in the working memory layer that are (2) encoded more accurately and (3) maintained more accurately through delays (i.e., less likely to drift, be interfered with, or “die out”); (4) increased accuracy of peaks, along with stronger associated inhibition, leads to more precise discrimination through a more robust comparison process; and (5) stronger activation and long-term memory traces support simultaneous encoding and maintenance of more items (i.e., increased capacity). Our simulations illustrate how this single developmental mechanism can account for a wide range of behavioral phenomena across tasks and domains.

## Modeling Applications Across Domains

In this section, we review prior research applying the DNF model to seven visuospatial tasks that span domains and developmental periods. Note that this review within these domains is not exhaustive; additional applications within these domains, as well as foundational modeling work that preceded the applications we discuss here, can be found in previous domain-specific reviews (Johnson and Simmering, 2015; Perone and Ambrose, 2015; Simmering and Schutte, 2015). Our goal is to present examples that best illustrate emergence, stability, and continuity. Each task the model simulates is described in boxes. The boxes describe the task, its intended use, typical age range, and general pattern of behavior observed over development. The boxes also provide a synopsis of the simulations to highlight the dynamics of the model involved in accounting for developmental change in performance (e.g., correct performance on the A-not-B task) or task-specific patterns of behavior (e.g., false alarms in visual change detection).

### Spatial Cognition

In this section, we describe the first applications of the DNF model to three types of spatial tasks that were previously accounted for by different theories of performance and development: the A-not-B task, spatial recall, and position discrimination.

#### A-not-B Task

In the canonical version of the A-not-B task (Piaget, 1954), shown in Box 2, an infant sits in front of a box with two wells. An experimenter hides a toy in one well (the A location) and the infant retrieves it after a short delay. Following a few trials hiding the toy at the A location, the experimenter hides the toy in the other well (the B location). The primary empirical question is whether they successfully search at B on the first B trial, or reach perseveratively to A, committing an A-not-B error. The main empirical finding is that younger infants (typically 8–10 months of age) make A-not-B errors, and by about 12 months of age, infants will typically reach correctly on B trials (for a review, see Marcovitch and Zelazo, 2009).

BOX 2 **Summary of Piagetian A-not-B task.**

**Task**. The Piagetian A-not-B task was developed as a measure of object permanence and is now considered to rely on working and long-term memory for location. The task is often used with 8- to 12- month-old infants. Infants are presented with a box with two hiding wells at locations designated as “A” and “B.” The task begins with a series of A trials on which an experimenter shows the infant a toy and then hides the toy in the well at the A location. The experimenter pushes the box toward the infant, and the infant normally reaches for the hidden object at the A location. After the A trials, the experimenter hides the toy at the B location. Typically, 10- to 12-month-old infants will correctly reach to the B location, whereas 8- to 10-month-old infants will reach perseveratively to the A location, making the A-not-B error.

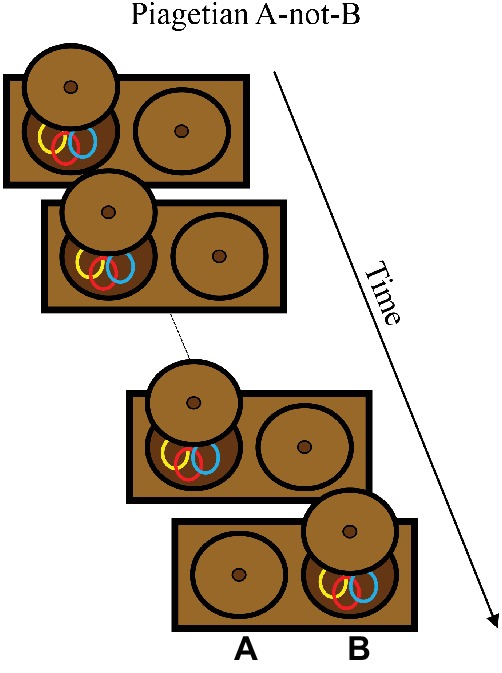

**Simulation Synopsis**. Where the model “reaches” in this task corresponds to the location of a peak in the working memory layer at the end of the delay. Across A trials, the model accumulates a memory trace at A. On the first B trial, the model with “older infant” parameters version is able to maintain a working memory peak at the B location, corresponding to a correct reach. With the “younger infant” parameters, by contrast, the model cannot maintain the working memory peak at B and instead forms a peak from the history at the A location, leading to the A-not-B error. Developmental change in the model is attributable to the Spatial Precision Hypothesis – stronger excitatory and inhibitory neural interactions – which enables the model to encode and maintain the hiding event at B.

Simmering et al. (2008) demonstrated that the DNF model could simulate infants’ performance in the A-not-B task, with accurate reaches on A trials, perseverative errors on B trials for younger infants, and correct reaches on B trials for older infants (for prior DNF model implementations, see Thelen et al., 2001). To simulate the task, the model is presented with constant weak inputs at sites tuned to locations A and B corresponding to the two wells, and a transient strong input at A or B to implement the toy being hidden. The model encodes the hiding event, building a peak at A in the working memory layer that remains active through the delay. To cue infants to search in the behavioral task, the apparatus is pushed forward to be within the infant’s reach; the analogous cue to the model is a boost to the inputs corresponding to the hiding wells. This boost raises activation associated with the locations of the two wells to near threshold levels. Activation at the A location has been strengthened from observing the hiding event, which leads to a strong peak in the working memory layer at this location. This peak is recorded as the location of the model’s reach (see Maruyama et al., 2014, for discussion of generating autonomous behavior from peaks in the context of A-not-B).

In Simmering et al.’s (2008) simulations, the model acquired a strong memory trace at the A location from repeated working memory peaks (and correct reaches) across trials (see Dineva and Schöner, 2018, for discussion of A-trial errors’ influence on B-trial performance). When tested on the first B trial, the model was unable to maintain a working memory peak at the B location. Over the delay in the trial, the memory trace from A effectively “knocked out” the peak at B, leading the model to instead form a peak at A, indicating a perseverative reach. These dynamics captured young infants’ performance well. Older infants’ performance was simulated by implementing the Spatial Precision Hypothesis. This improves the stability of the working memory peaks which, in turn, enables the model to maintain items in working memory for longer durations by resisting interference and spontaneous decay. In this “older” model, the hiding event at B was encoded and maintained as a stable peak in the working memory layer through the delay, and the model generated a correct response to B.

Spencer et al. (2001) hypothesized that these same processes at work in the A-not-B task would be evident in older children if the task structure was changed to be more sensitive to fluctuations in memory. They tested 2-year-olds’ memory for spatial locations in a homogeneous sandbox using an A-not-B design and found that, in the continuous space that allowed for search errors in between the A and B locations (which were not marked in the sand), toddlers’ searches on B trials was biased toward the A locations. Schutte et al. (2003) then extended these findings later in development, showing the same phenomenon in 4- and 6-year-old children, although only when the distance between A and B was sufficiently small. These studies using the sandbox task indicate continuity in processes at work in the A-not-B task with to a second domain, spatial recall, as well as development from infancy to early childhood.

#### Spatial Recall

Children’s memory for spatial locations in continuous spaces can also be studied in the “spaceship” task. The task minimizes the visual cues available, and hiding locations are typically distributed throughout the space to reduce the influence of long-term memory. Box 3 shows the “spaceship” task in which children sit in front of a homogenous-surfaced table. On each trial, a small target “spaceship” appears briefly, then disappears for a short delay, after which the child points or places a rocket marker on the remembered location (Schutte and Spencer, 2002). As in the sandbox task, participants’ searches are biased toward previously-remembered locations (Schutte and Spencer, 2002; Spencer and Hund, 2002; Lipinski et al., 2010). In addition, participants show delay-dependent errors in these tasks, with small errors after short delays and increasingly larger errors at longer delays; with age, the overall magnitude of errors decreases. These errors are systematically biased in these task spaces, with younger children (approximately 2–4 years) recalling locations as closer to the center of the space (bias toward the midline symmetry axis) and older children (5–11 years) and adults recalling locations as farther from the center of the space (bias away from midline; e.g., Spencer and Hund, 2003). The distance of the hiding location from the edges and midline symmetry axis also influences the magnitude and direction of errors (Schutte and Spencer, 2009).

BOX 3 **Summary of spatial recall.**

**Task**. The spatial recall task measures location memory in a uniform space. Variations of this task have been used with children as young as 18 months and adults. On each trial, a small target (see pink star) briefly appears, disappears for a short delay, and the child is asked to point to the remembered location (see arrow location). The task briefly appears, disappears for a short delay, and the child is asked to point to the remembered location (see arrow location). The task requires recalling the location a stimulus appeared and a behavioral response at the remembered location. A central feature of this task is its minimal perceptual structure and the possibility of presenting stimuli across the full extent of the task space. Children and adults exhibit delay-dependent errors in these tasks, with error magnitude increasing with longer delays. These delay-dependent errors are systematically biased such that 2- to 4-year-old children recall locations as closer to the center of the space and 5- to 11-year-old children and adults recall locations as farther from the center of the space. Children and adults also show biases toward previously remembered locations in the task space. The magnitude of errors, including the spatial span across which previous experience influences performance, generally decreases over development.

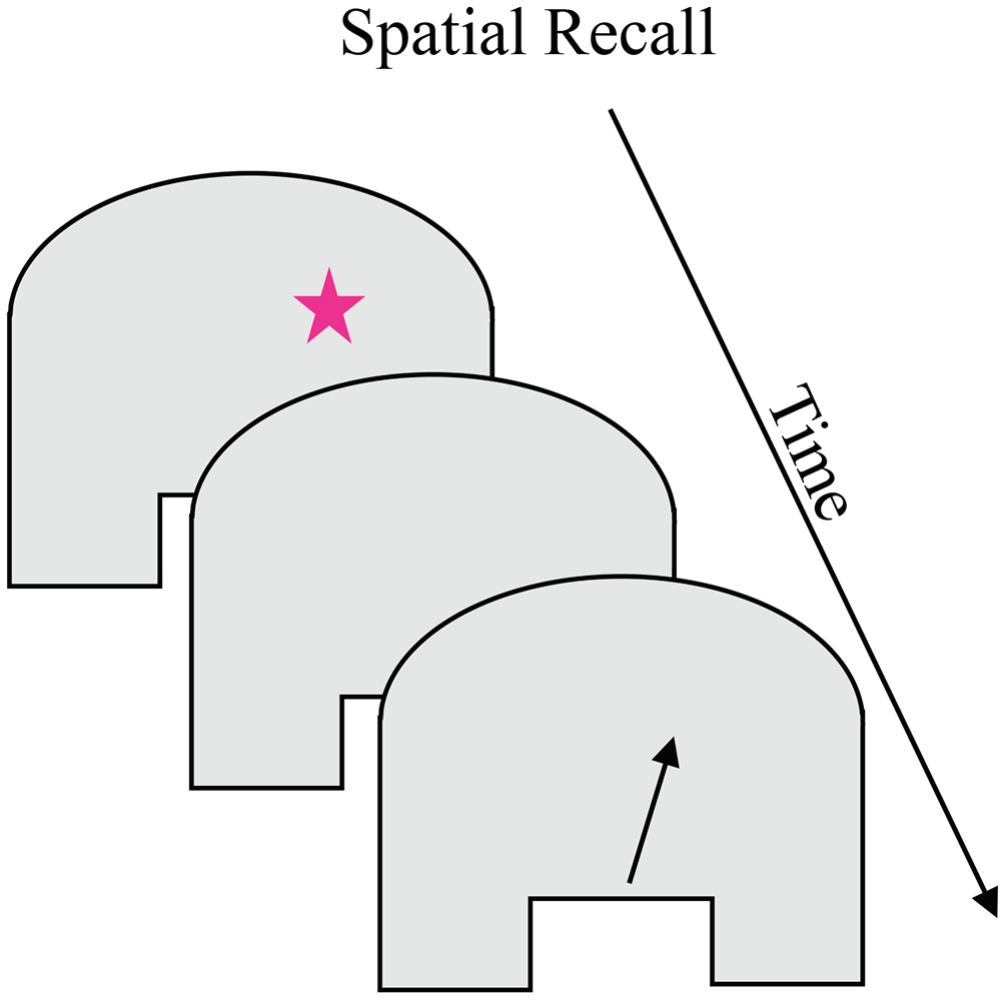

**Simulation Synopsis**. For simulations of young children’s performance, weak neural interactions result in broad excitation associated with the midline symmetry axis. The broad excitation of the midline symmetry axis attracts the working memory peak for target location during the delay. This causes the working memory peak for location to drift toward midline. At the end of the delay, the location of that peak is read out as the model’s pointing response which is closer to midline than the target location. For simulations of older children’s performance, stronger neural interactions create strong inhibition surrounding the symmetry axis input in the working memory layer. This inhibition repels the working memory peak for target location. When the location of the peak is read out at the end of the delay, it is farther from midline than the target location. In parallel to changes in the direction of drift, the increasing stability of peaks in the working memory layer leads to a general decrease in error magnitude.

Simulations of children’s performance in the spaceship task were a natural extension from A-not-B by adapting the perceptual input corresponding to the task space: removing the two specific task inputs (hiding wells) and adding an input to implement the midline symmetry axis. Schutte and Spencer (2009) showed that the neurocognitive processes in the DNF model could produce the complex pattern of performance that children exhibit between the ages of 3 and 5 years, shifting from bias toward midline to bias away from midline differentially across the target distances from midline. They situated the model in the “spaceship” task by providing a relatively weak input for the midline symmetry axis, and a strong input for the presentation of the spaceship (see Schutte et al., 2011, for simulations of the sandbox task). With weaker interactions early in development, the midline symmetry axis results in a small excitatory boost at that central location. When the target is presented, it forms a peak in the working memory layer; after input is removed, the peak is maintained through local interactions. Critically, the position of this peak during the delay is influenced continuously by the presence of midline in the task space. The excitatory activity associated with midline attracts activation, which causes the peak to drift toward midline over time. At the end of the delay, the location of that peak is read out as the model’s pointing response. The “young” model shows the same pattern of bias as young children, recalling locations as closer to midline than they really were. Implementing the Spatial Precision Hypothesis to create an “older” model led to the midline symmetry axis being more precisely encoded. This produced an inhibitory influence surrounding the central location, resulting in the peak in the working memory layer being “pushed” or repelled during the delay.

In relation to the A-not-B simulations, the spatial recall simulations accounted for bias through the real-time interactions between representations: in this case, the midline symmetry axis influenced the location estimated in the working memory layer on the current trial, rather than the memory trace (see Lipinski et al., 2010, for simulations of both influences simultaneously). Both tasks also connect memory processes to reaching or pointing behaviors, showing how the specific patterns of errors emerge from the interaction of cognitive processes with the physical structure of the task space (presence versus absence of hiding wells). Spencer et al. (2006) illustrated a similar point in a task with 7-year-old children and adults, in which participants indicated a remembered location either by drawing an X or choosing from a set of dots. Results showed that children’s biases were under-estimated on choice trials if the dots did not overlap with where their memory had drifted; that is, drawn responses were farther from the target location than any of the choice options, so children’s selections appeared more accurate because they did not reflect the true drift in memory. Thus, the structure of the task, in the spatial span of the choice locations, modulated whether biases appeared similar across response types.

Another important feature of these spatial recall simulations is they highlight how ongoing processes influence behavior. Interactions between representation of midline and location occur continuously through the delay: as soon as the stimulus input is removed, the interactions begin to induce bias. This feature has implications for a competing theory of recall biases, the Category Adjustment Model (Huttenlocher et al., 1994), in which biases result from combining spatial category information and fine-grained location information. In that account, this combination weighs more heavily toward category information due to uncertainty in fine-grained information that results from the delay. Thus, biases should not appear when delays are short because uncertainty should be low. As the next section illustrates, however, delay-dependent biases can be detected at very short delays when tested in a different paradigm, position discrimination.

#### Position Discrimination

The position discrimination task was developed to test perceptual resolution across spatial layouts in adults (e.g., near or far from other stimuli; Palmer, 1986). Simmering and Spencer (2008) modified the task for use with children to test predictions inspired by the Spatial Precision Hypothesis account of development in the spatial recall task. Box 4 shows the position discrimination task. Participants are situated at a table with a perceptually uniform surface (c.f., the “spaceship” task described above) and a stimulus appears briefly at one location, disappears for a short delay, and then reappears and the participant judges whether the two positions were “same” or “different”. Individual and developmental differences in the distance necessary to indicate “different” [sometimes termed just-noticeable difference (JND)] can be evaluated by manipulating the distance and direction between the two stimuli.

BOX 4 **Summary of position discrimination.**

**Task**. The position discrimination task was developed as a test of perceptual resolution (how small of a distance between stimuli can be detected) in adults and has been used with children between 3 and 6 years of age. Children are situated at a table with a perceptually uniform surface and a stimulus briefly appears at one location. Children are situated at a table with a perceptually uniform surface and a stimulus briefly appears at one location, disappears for a short delay, and then briefly reappears. The child responds whether the two positions were “same” or “different.” The just-noticeable difference (JND) is computed which is the distance necessary to reliably indicate “different” and can be evaluated across individuals and ages by manipulating the distance and direction between the two stimuli. JNDs decrease between the ages of 3 and 6 years. When the second stimulus is presented in the direction of bias seen in spatial recall (i.e., toward the center of the space for younger children, away from the center of the space for older children and adults), JNDs are larger than when the stimulus is presented in the opposite direction. The figure shows the presentation of the first stimulus (smiley face), followed by a delay, and finally the presentation of the second stimulus. The left panel shows the second stimulus appear closer to midline; the right panel shows the second stimulus appear farther from midline.

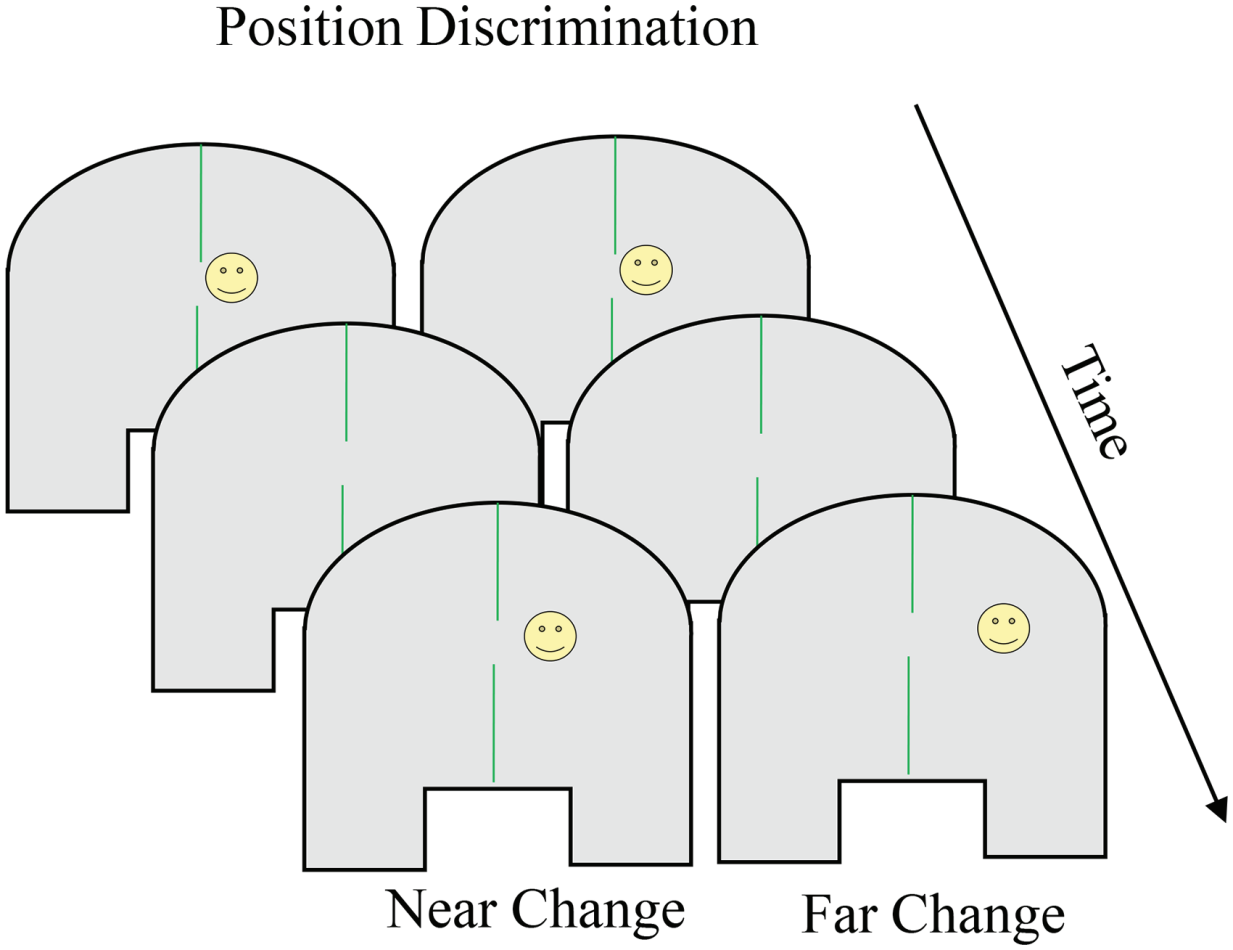

**Simulation Synopsis**. The model’s comparison of the first stimulus, represented as a peak in the working memory layer, and the second stimulus, represented as new input to the contrast layer, depends on the inhibitory connection between layers. When the location of the second stimulus and the location of the first stimulus held in the working memory layer overlap substantially, activation from the working memory layer projects to the same node and a “same” response is generated. When the second stimulus does not overlap with the location of the first stimulus held in the working memory layer, activation in the contrast layer projects to the different node and a “different” response is generated. The spatial asymmetry in JNDs results from drift of the peak in the working memory layer during the delay, driven by the midline symmetry axis as in spatial recall. Developmental reductions in JNDs result from stronger and more accurate peaks in the working memory layer and the associated inhibition that projects to the contrast layer.

Research with adults established smaller (i.e., more accurate) JNDs near visible lines and symmetry axes (e.g., Palmer, 1986). Simmering and Spencer’s (2008) showed changes in JND and bias (based on the direction of the second dot relative to the first) during childhood that mirror changes in spatial recall. In particular, JNDs were larger (i.e., less accurate) earlier in development, decreasing between the ages of 3 and 6 years then into adulthood, and when the second stimulus was presented in the direction of bias seen in spatial recall (i.e., toward midline for young children, away from midline for older children and adults), JNDs were larger than when the stimulus was presented in the opposite direction (Simmering and Spencer, 2008). Simmering and Spencer (2008) predicted JNDs would decrease while the direction of bias shifted over development based on the mechanisms inherent in the simulations Schutte and colleagues used to capture spatial recall performance (see Simmering and Schutte, 2015, for review).

To simulate position discrimination in DNF model, two critical differences from recall tasks needed to be addressed. First, a second stimulus position had to be compared to the memory representation of the first stimulus. Second, a decision of “same” or “different” had to be made based on that comparison. The comparison processes emerges from the cross-layer interactions within the neurocognitive system: when the second stimulus overlaps spatially with the item in memory, inhibition suppresses excitation in the contrast field; when it does not overlap, excitation in the contrast field corresponds to the encoding of the new item. To generate a response from this process, the decision system shown in Box 1 was coupled to the neurocognitive system. When the second stimulus in the task space does not overlap with first stimulus held in working memory, strong excitation in the contrast layer activates the “different” node to suprathreshold levels. This corresponds to a “different” response. When the second stimulus overlaps with the first stimulus held in working memory, strong excitation in the working memory layer activates the “same” node which corresponds to a “same” response. Simmering and Spencer (2008) used this relatively simple response system to illustrate the influences of both the distance and direction of the second stimulus over development in position discrimination.

#### Synthesis of Spatial Cognition

Across these three spatial cognitive tasks, the emergence is illustrated through the difference in behavioral signatures driven by the task structure. Bias toward previously-remembered locations in the A-not-B and sandbox tasks emerges through each individual participant’s history in the task (Dineva and Schöner, 2018). The A-not-B error seems to disappear by 12 months of age in the context of the distinct hiding locations in the A-not-B task, but when memory is probed in continuous space with variable distances between hiding locations, similar biases are evident throughout childhood and into adulthood (Spencer et al., 2001; Schutte and Spencer, 2002; Spencer and Hund, 2002; Schutte et al., 2003; Lipinski et al., 2010). When locations are recalled within different tasks spaces, reference-related biases emerge through interactions of memory with perceptual structure (Schutte and Spencer, 2009, 2010; Schutte et al., 2011) and the influence of memory biases appears different when the response is to select from choices (Spencer et al., 2006) or generate a same/different response (Simmering and Spencer, 2008) rather than pointing to a remembered location.

Stability accounts for both real-time and developmental phenomena across these tasks. In the A-not-B task, a history of experience leads to more stable reaches on A trials, which cannot be overcome by the memory of B for young infants. Older infants also establish a history at A, but through stronger interactions supporting memory, they can more quickly form a stable representation of B to support a correct reach to B. Increasing stability in the maintenance of memory representations accounts for developmental reductions in the magnitude of errors in spatial recall (Schutte and Spencer, 2009) and JNDs in position discrimination (Simmering and Spencer, 2008). Less stable representation of symmetry leads to a weak attractive influence on memory, as seen in young children’s bias toward midline in recall and poorer discrimination of changes in the direction of midline in position discrimination. As reference frames are represented more stably in real time, the influence shifts to repulsion through inhibitory interactions, leading to transitions in the direction of these effects.

Continuity is also evident in the spatial cognition simulations. The ongoing real-time processes required to encode items and maintain representations in memory serve an emergent comparison process in the position discrimination task when coupled to a same/different decision system. The continuous drift of peaks in the working memory layer underlies delay-dependent biases in spatial recall and asymmetries in position discrimination. And the same ongoing processes are at work across simulations of the spatial cognition tasks and development.

In the next section, we describe the application of the DNF model to tasks in domains requiring comparison. The DNF model simulations of position discrimination showed how comparison emerges in a task context in which an item is maintained in memory while a novel item appears in the task space. This breakthrough formalized recognition of familiarity and detection of novelty that enabled the DNF model to be extended into two new domains simultaneously: infant visual exploration and visual working memory (VWM). The DNF model used in the simulations consisted of layers tuned to continuous feature (e.g., color) dimensions (as shown in Figure 1).

### Infant Visual Exploration

The ability of the model to compare visual stimuli connects to classic paradigms in infant visual exploration. Much of what is known about infant visual exploration is based on the observation that infants’ looking time to a stimulus declines as it becomes familiar and increases to a discriminably different stimulus. This dwindling interest in the familiar stimulus has long been attributed to memory formation and recognition, whereas interest in the novel stimulus is thought to reflect renewed attention and encoding into memory. This basic process is already at work in the DNF model. When a stimulus is presented as an input to the model, the item is encoded in the contrast layer into the working memory layer. A robust working memory peak, facilitated by long-term memory formation, inhibits further encoding of the familiar item – recognition. A new item excites uninhibited neurons in the contrast layer – novelty detection. Applying the DNF model to infant visual exploration required equipping it with a fixation system to specify the link between looking and the neurocognitive processes in the model. The fixation system, shown in Box 1, is coupled to the contrast layer and can look at left, center, and right locations at which stimuli appear, as well as away locations at which no task relevant stimuli appear. The presence of items in the task bias the fixation system to gaze in their direction. This biasing can be construed as perceiving locations that afford action – looking, in this case – in the environment. When the model looks at a location in the task space, the stimulus from that location is input to the contrast layer and the stimulus is encoded into the working memory layer. Activation associated with encoding sustains the looking node at suprathreshold levels, accumulating looking time. As the peak associated with the stimulus in the task space is formed in the working memory layer, activation associated with encoding the item in the contrast layer is suppressed and fixation to the stimulus location is released. With the addition of the fixation system, the DNF model can be used to understand infants’ performance in single presentation habituation and visual paired comparison.

#### Habituation

The habituation paradigm (Cohen, 1969) relies on a decrease in looking behavior as an index of memory formation for a stimulus, and renewed looking to a new stimulus as an index of discrimination between the remembered and new stimulus. Box 5 shows the habituation paradigm, which consists of habituation and test phases. During the habituation phase, infants are repeatedly presented with a single stimulus (blue star) to which their looking time declines across trials. During the test phase, memory for the stimulus can be probed across a series of trials. With age, infants exhibit a faster decline in looking to the familiar stimulus and are able to discriminate between increasingly subtle stimuli (for review, see Colombo and Mitchell, 1990).

BOX 5 **Summary of infant habituation.**

**Task**. The habituation paradigm has been used to measure memory formation and discrimination for visual stimuli during infancy. During the habituation phase, infants are repeatedly presented with a single stimulus (e.g., blue star) to which their looking time declines across trials. During the test phase, memory for the stimulus can be probed for different features of the object (e.g., color, shape) or the whole object (e.g., color and shape). With age, infants exhibit a faster decline in looking during the habituation phase and are able to discriminate between highly similar stimuli. The figure shows presentation of a single stimulus across a series of habituation trials followed by novel stimuli presented across a series of test trials.

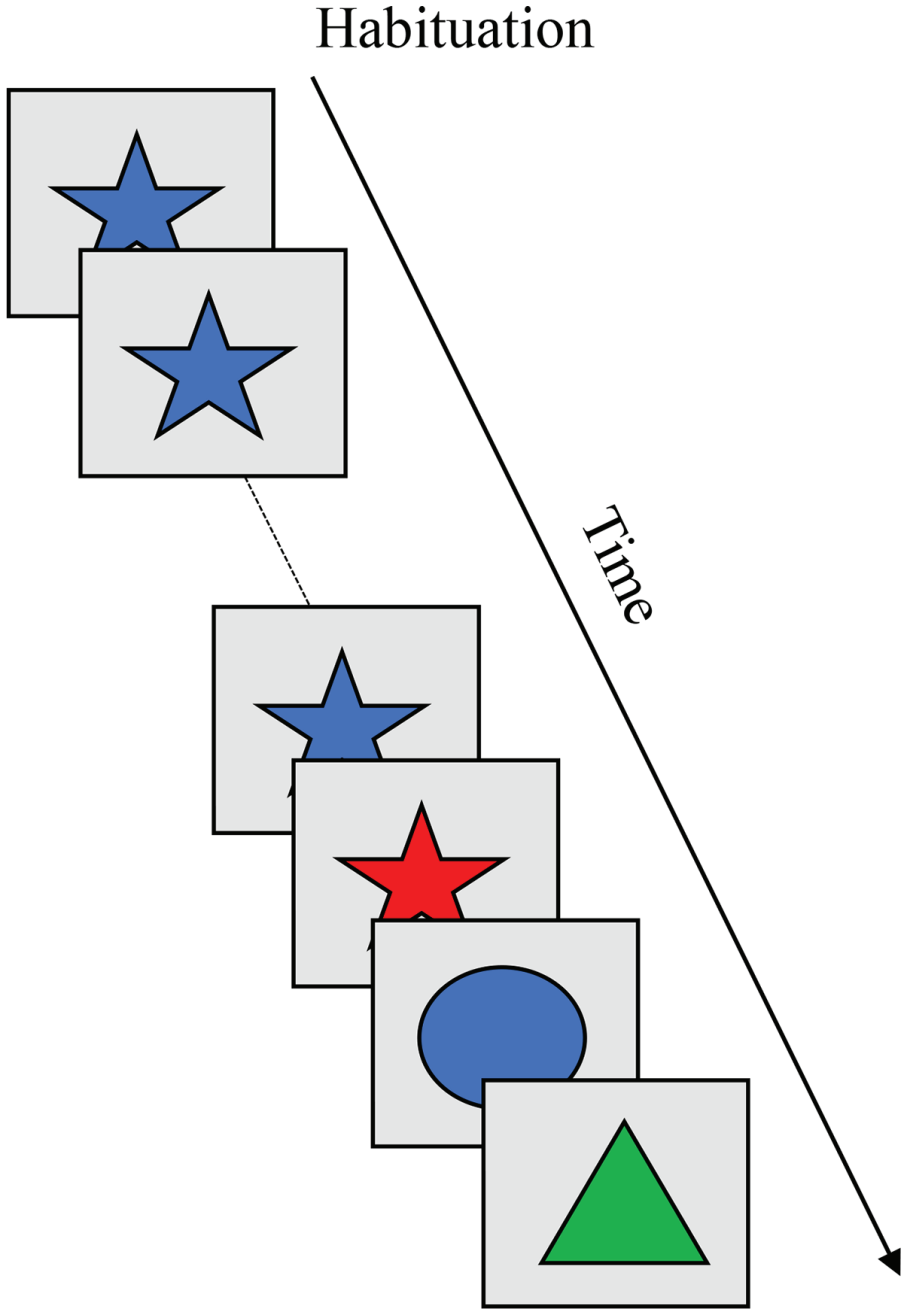

**Simulation Synopsis**. Across a series of trials, a stable peak in the working memory layer is formed. This suppresses similarly tuned neurons in the contrast layer, releasing the looking node and leading to a decrease in looking time. During the test phase, the model is presented with novel items that excite uninhibited neurons in the contrast layer which sustains the looking node. The model is said to discriminate between the remembered and novel stimuli. Developmental change in habituation rate and discrimination is attributable to stronger neural interactions, which enables the model to rapidly form a stable working memory peak and, in turn, faster habituation of looking. Stronger excitation in the contrast layer enables it to respond to stimuli that are similar to those held in the working memory layer.

Perone and Spencer (2013b) situated the three-layer DNF model in the habituation paradigm to provide an account of canonical patterns of developmental change in habituation rate and discrimination. Their central hypothesis was that habituation involves the same neurocognitive dynamics as spatial cognition, but the influence on looking behavior happens more slowly as the model transitions from an encoding state in the contrast layer to a peak state in the working memory layer across repeated presentations of a stimulus (c.f., Figure 1). Early in the habituation phase, the model primarily encodes the stimulus which drives high levels of looking. As the model acquires memory traces for the stimulus, a peak in the working memory layer emerges. This suppresses encoding through the same recognition process that drives “same” responses in positions discrimination. In the context of a looking paradigm, however, the suppression of encoding releases fixation and leads to low levels of looking. During the test phase, the model is presented with novel items that vary in similarity to the remembered item. The model dishabituates when the novel and remembered items are dissimilar but not when they are similar. When the Spatial Precision Hypothesis is implemented in the model, the working memory layer more quickly acquires a peak, habituating more quickly. The contrast layer also responds strongly to novel items that are similar to the remembered item, leading to high levels of looking. With development the model, like infants, exhibits a faster decline in looking and can discriminate between highly similar familiar and novel items.

The DNF model simulations also specified a link between the act of looking and learning. Previous conceptual and computational models had emphasized the influence of the representations on looking (Cohen, 1972a,b, 1973; Hunter and Ames, 1988; Sirois and Mareschal, 2004). The reciprocity between looking and learning has been demonstrated in the visual paired comparison paradigm (Jankowski et al., 2001), which we highlight in the simulations described next.

#### Visual Paired Comparison

The visual paired comparison (VPC) task (Fagan, 1970) is similar to the habituation paradigm but with stimuli presented in pairs. Box 6 shows the familiarization and test phases in VPC. During familiarization, infants are presented with pairs of identical items across a series of trials (blue star). The familiarization phase ends after a set number of trials or once the infant accumulates a predetermined amount of looking time. During the test phase, the familiar item is presented with a novel item (blue star paired with a red star). A novelty preference (significantly more looking to the novel item) is taken as evidence that infants recognize the familiar item. VPC yields a rich set of looking dynamics, including shift rate (gaze switches relative to time spent looking), average look duration, and peak look duration. Rose et al. (2001) showed with age, infants exhibit stronger novelty preferences, faster shift rates, and shorter look durations (both average and peak). Individual differences in these looking behaviors during familiarization predict the strength of novelty preferences during the test phase. For instance, infants who exhibit higher shift rates and shorter look durations also exhibit stronger novelty preferences.

BOX 6 **Summary of visual paired comparison (VPC).**

**Task**. VPC is used to measure recognition memory during infancy. Infants are presented with pairs of identical items across a series of trials (blue star). The familiarization phase ends after a set number of trials or once the infant accumulates a predetermined amount of looking time. During the test phase, the familiar item is presented with a novel item (blue star paired with a red star). A novelty preference (significantly more looking to the novel item) is taken as evidence infants recognize the familiar item and are processing the novel item. VPC yields a rich set of looking dynamics, including shift rate (gaze switches relative to time spent looking), average look duration, and peak look (longest look duration). With age, infants exhibit higher novelty preferences, faster shift rates, and shorter look durations. Individual differences in these looking behaviors during familiarization predict the strength of novelty preferences during the test phase. For instance, infants who exhibit higher shift rates and shorter look durations also exhibit stronger novelty preferences.

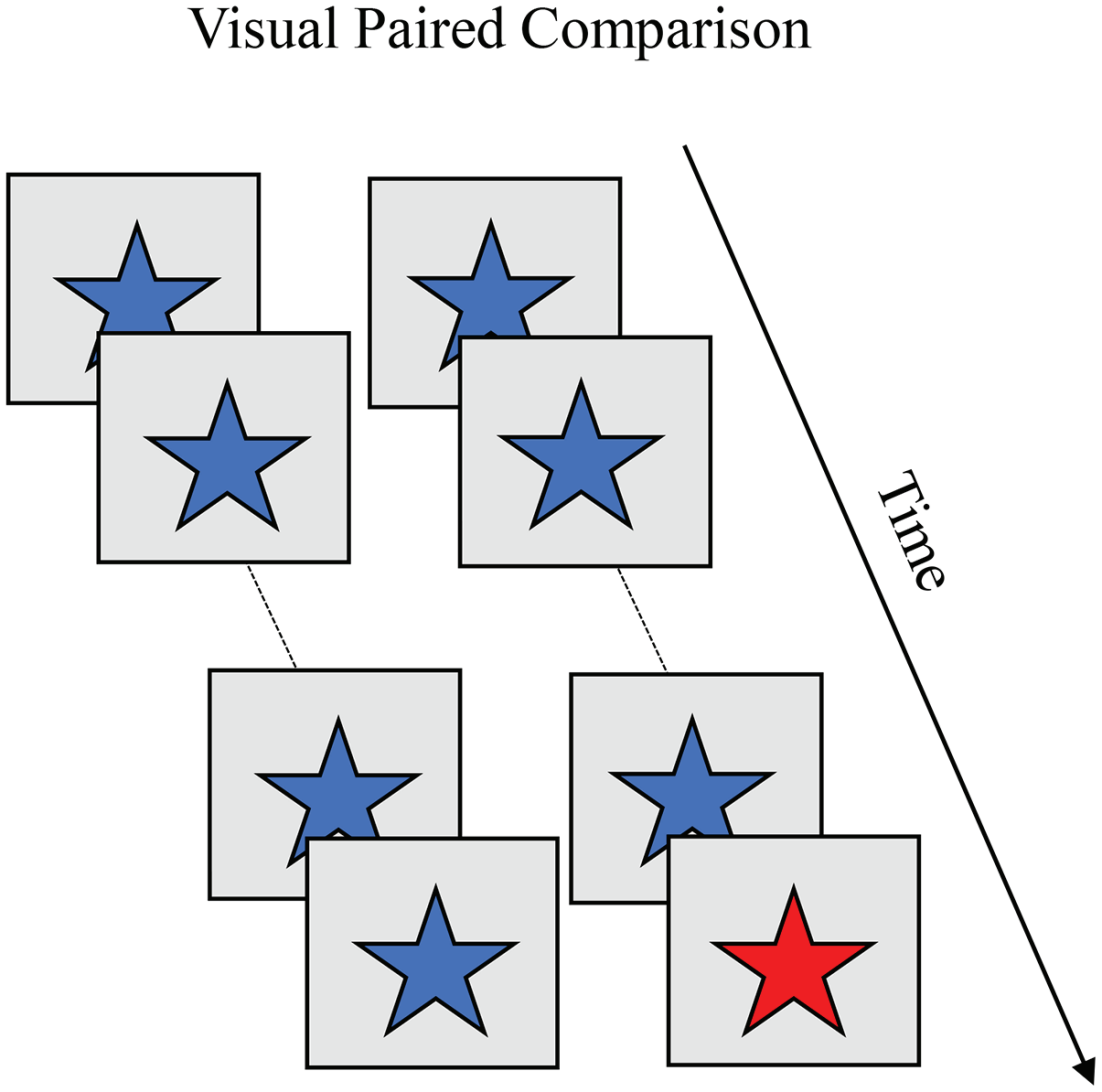

**Simulation Synopsis**. A stable working memory peak for the familiarization items emerges more quickly with stronger neural interactions. This leads to faster recognition of the familiar items and faster release of fixation. The result is shorter look durations and more shifting with age. The autonomous nature with which the model explores pairs of identical items during familiarization leads to simulation-to-simulation variation in the rate at which a stable working memory peak emerges for the familiar stimulus. Simulations that more quickly form working memory peaks during familiarization exhibit shorter look durations, more shifting, and, at test, stronger novelty preferences.

Perone and Spencer (2014) situated the DNF model in the VPC in which identical items appeared during the familiarization phase, and the familiar item paired with a novel item during the test phase. There were two influences on looking. One influence was stochastics: the presence of two items sometimes made the model switch gaze. The other influence was the Spatial Precision Hypothesis: the stronger the interactions in the model, the faster it encoded items into working memory. When the model had encoded the items into the working memory layer and fixated one of the now familiar items, it quickly switched gaze. Thus, noise and working memory formation both influenced look duration and gaze switching.

Perone and Spencer (2014) used the model to account for developmental change in infants’ looking dynamics and discrimination between 5 and 10 months of age. They showed that the model, like infants, exhibited a faster shift rate, shorter look durations, and shorter peak looks with age. They also showed that the model, like infants, could discriminate stimuli along continuous metric dimensions (e.g., color) with age. The theoretically interesting observation was individual differences in looking dynamics during the familiarization phase predicted novelty preferences during the test phase in infants and the model *even though there were no individual differences built into the model.* This suggests looking dynamics themselves play a role in structuring learning. Previous views on individual differences in looking attribute them only to differences in cognitive processing (Rose et al., 2012).

Looking in the DNF model is consistent with the view put forth by Reynolds and Romano (2016) that infants’ emerging attentional abilities are critical for performance in working memory tasks. In the DNF model, building stable peaks in the contrast layer supports fixation which, in turn, allows encoding into working memory. Without building robust peaks in the contrast layer, there is no sustained looking and, in turn, no capacity to ignore looking at distractors. Without building robust peaks in the contrast layer, there is no capacity to acquire a working memory, characteristic of younger infants (for review, see Reynolds and Romano, 2016; see Perone and Spencer, 2013b; for exemplary simulations of Wetherford and Cohen, 1973). In the DNF model, infants’ developing control over attention, and capacity to form working memories, emerges from the stability of peaks involved in encoding stimuli into working memory.

#### Synthesis of Infant Visual Exploration

Connecting the simulations from spatial cognition with those in infant visual exploration provides additional illustration of the importance of emergence, stability, and continuity in understanding behavior, cognition, and development. Habituation and VPC rely on the same underlying cognitive processes of encoding, maintenance, and comparison that were evident in the spatial cognition simulations, but the coupling of looking dynamics with memory formation lead to emergent consequences of the task structures. With only one stimulus presented repeatedly, looking time decreases in the canonical habituation pattern until a new stimulus is presented and looking increases (dishabituation). With two identical items presented simultaneously, preferences emerge through the bi-directional interplay of looking between the two stimuli and memory formation. In fact, individual differences in looking can emerge from spontaneously dwelling on a stimulus for extended duration, strengthening memory formation and subsequent shortening look durations.

Stability plays a central role in looking. The formation of a stable peak in the contrast layer sustains looking and makes encoding stimuli into working memory possible. The stability of working memory peaks enables faster working memory formation, leading to faster release of fixation. These simulations showed increases in the stability of working memory can account for they key phenomena in looking tasks also provides continuity across domains and development, as they are the same general processes that explain transitions in A-not-B errors during infancy and recall biases and position discrimination during childhood. The application of the DNF model to infant visual exploration made the application to VWM capacity in infancy possible, which we describe next.

### Visual Working Memory Capacity

A hallmark of VWM is its limited capacity, estimated to be only 3–5 simple items in adults (e.g., Cowan, 2010) and fewer in children (see Simmering, 2016, for review). Why VWM capacity is limited has been a long-standing source of debate. For decades, the dominant account of capacity limits was a slot-like characterization, in which a small number of discrete objects could be stored (e.g., Vogel et al., 2001). One challenge to this account came through evidence that the resolution or precision of memory depended on load, suggesting that a pool of resources distributed across objects was a more apt characterization (e.g., Bays et al., 2009). In the DNF model, the number of items held in memory is limited by the real-time neurocognitive processes that support encoding and maintenance – the same processes that accounted for phenomena in spatial cognition over development and infant visual exploration. We begin by describing the infant change preference task, followed by the visual change detection task that it was modeled after.

#### Infant Change Preference Task

The change preference task, shown in Box 7, was designed to probe VWM capacity during infancy (Ross-Sheehy et al., 2003). The task involves two simultaneous displays, a no-change and a change display, in which colored squares blink on and off over the course of a trial. Colors on the no-change display remain the same across blinks while, on the change display, one color changes on each blink. The logic is if the infant can remember the items on the display across the delay, they should find the novelty of the change display more interesting to look at than the familiarity of the no-change display. A change preference is calculated as the proportion of time fixating the change display out of total time fixating both displays. Age-related change in VWM capacity can be assessed by manipulating the set size (i.e., number of items on the display), and capacity is estimated as the highest set size at which infants show a reliable change preference. Ross-Sheehy et al. (2003) found that infants’ VWM capacity increased from one to three or four colors between 6 and 10 months of age. These findings suggest that infants’ VWM capacity reached adult-like levels by the end of the first year. Further studies showed that, by 7.5 months, infants could remember three color-location correspondences.

BOX 7 **Summary of change preference.**

**Task**. The change preference task was designed to measure visual working memory (VWM) capacity during infancy. Infants are simultaneously presented with two displays, a no-change (left) and a change display (right). In the canonical variant, colored squares blink on and off over the course of a trial. Colors on the no-change display remain the same, while one color changes on each blink on the change display. The logic is if the infant can remember all the items on the display, they should notice the novelty of the change display and exhibit a change preference (more looking to the change than the no- change display). Age-related change in VWM capacity can be assessed by manipulating the set size (i.e., number of items on the display), and capacity is estimated as the highest set size at which infants show a reliable change preference. Infants’ VWM capacity increases from one to three or four colors between 6 and 10 months of age.

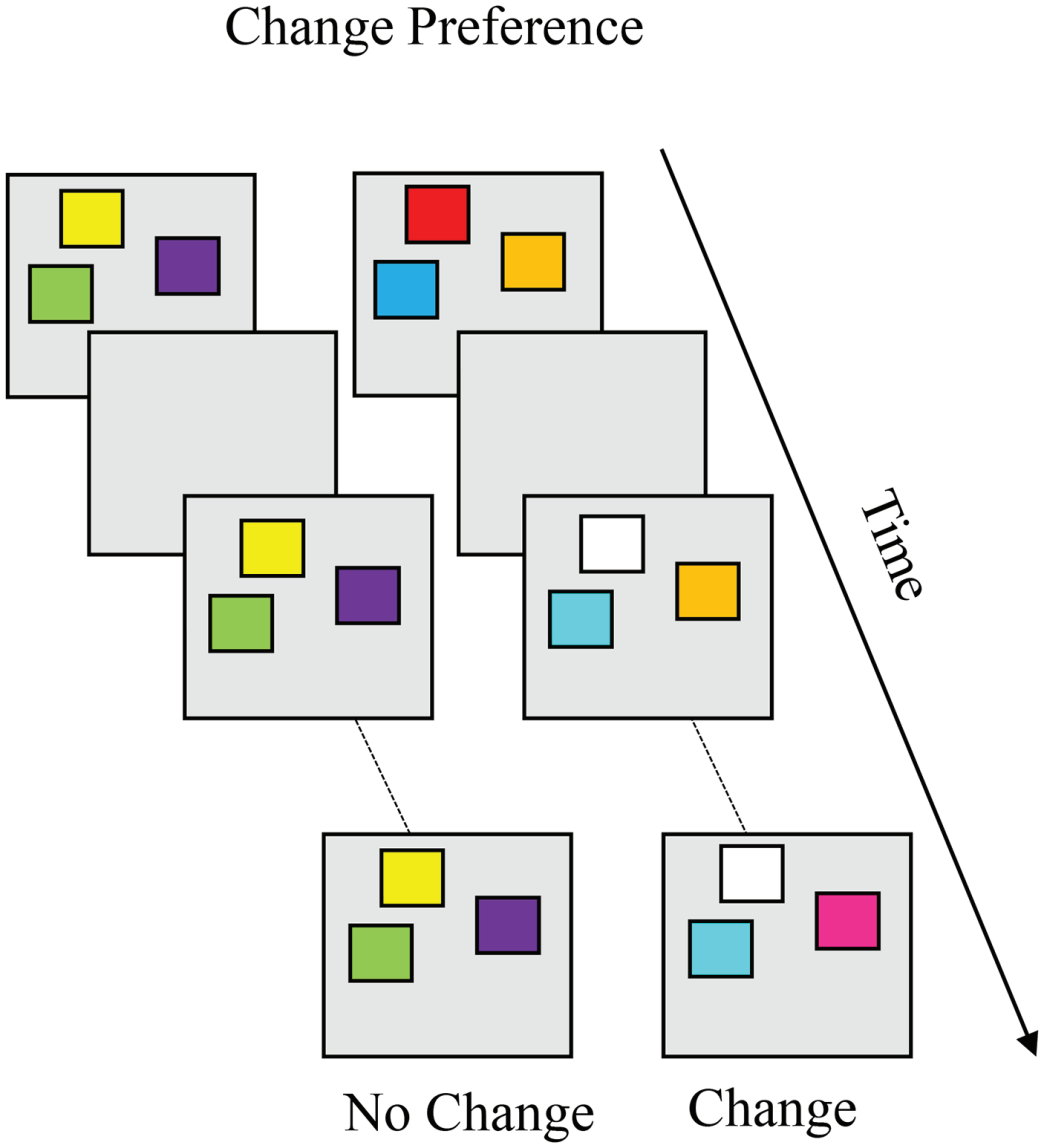

**Simulation Synopsis**. The model exhibits a change preference when activation in the contrast layer associated with the change display is stronger than activation associated with the no-change display. When the model acquires stable working memory peaks for the items on the no-change display, looking time to the no-change display is low. When the model looks at the change display, activation in the contrast layer generated by the changing items sustains the looking node. Stronger neural interactions lead the model to maintain more stable working memory peaks for the no-change display and, in turn, with age the model exhibits a change preference at higher set sizes.

Perone et al. (2011) situated the model in the same procedure as infants in Ross-Sheehy et al. (2003). For the model, performance depended critically on representation of the no-change display. If the model could recognize the no-change display as familiar, it was more likely to explore the change display where the presence of a novel item on each blink helped sustain looking. This, in turn, could lead to a change preference. Alternatively, if the model was unable to maintain the items from the no-change display, then the same colors would appear novel across presentations, leading to more equal looking across the change and no-change displays. Thus, the processes that drove a change preference were the same processes that drove dishabituation in the habituation paradigm and a novelty preference in VPC.

In order for the model to recognize the no-change display as familiar, at least some of the items must be encoded into working memory. This is where development came into the mix. Perone et al. (2011) implemented the Spatial Precision Hypothesis in the model, which led the model to form peaks in the working memory layer that were able to resist interference from incoming items being encoded. This enabled the model to recognize no-change displays containing more and more items with development, effectively boosting the model’s capacity. It also led to more robust novelty detection and, consequently, sustained looking at the changing display. Thus, capacity estimates in the task arise from robust peaks in the working memory layer and strengthened encoding. Perone et al. (2011) found that, in set sizes where it exhibited a robust change preference, the DNF model only maintained about half of the items on the no-change display in the working memory layer, and less than half of the items on the change display. This difference in memory for items from the two displays is what led the model to exhibit a preference for the change display (where more items were novel). More interestingly, the number of items that needed to be maintained to exhibit a change preference was considerably lower than the number presented on the display.

#### Visual Change Detection

The visual change detection task that inspired the change preference task was originally developed for use with adults (Pashler, 1988), but has been adapted for use from early childhood through adolescence (e.g., Cowan et al., 2005; Riggs et al., 2006; Simmering, 2012; Isbell et al., 2015). Box 8 shows the whole-array version of the change detection task, in which the same number of items are presented in the memory and test array. On each trial, participants are presented with a memory array with a small number of simple objects, then following a short delay a test array is presented, and the participant identifies whether the objects in the memory and test arrays were “same” or “different.” On no-change trials, these arrays are identical, and on change trials, arrays differ by one item. Capacity can be estimated using a formula that takes into account different response and trial types across set sizes (Pashler, 1988; see Cowan et al., 2005, for a variation for single-item test). The key phenomena to account for are capacity limits increase throughout childhood.

BOX 8 **Summary of change detection.**

**Task**. The change detection task is used to measure visual working memory (VWM) capacity in children and adults. On each trial, participants are presented with a memory array with a small number of simple objects (e.g., colored squares), then following a short delay a test array is presented. The participant identifies whether the objects in the memory and test arrays were “same” or “different”. On no-change trials, these arrays are identical, and on change trials, arrays differ by one item. Response types include hit (correct change), correct rejection (correct same), miss (report no change when change is present), and false alarm (report change when no change occurred). Capacity can be estimated using a formula that accounts for these response types across set sizes. Capacity estimates increase from about 1.5 items at 3 years to 3 items at 5 years to about 4.5 items in adults.

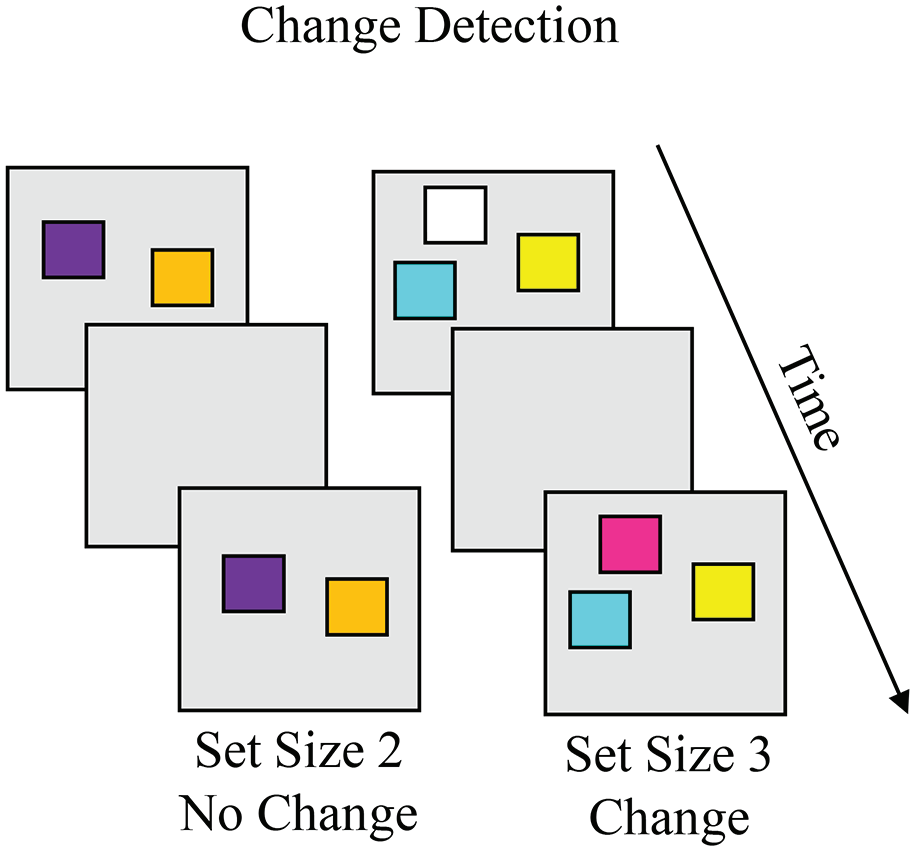

**Simulation Synopsis**. On change trials, the new item in the test array generates suprathreshold activation in the contrast layer, which projects to the different node to generate a “different” decision. On no-change trials, the items in working memory suppress locations tuned to those items in the contrast layer. Activation in the working memory layer projects to the same node to generate a “same” decision. With development, stronger neural interactions enable the model to encode more items in the working memory layer and maintain the peaks more stably over the delay which, in turn, leads the model to respond more accurately at higher set sizes. The model also accounts for the types of errors children and adults make. For instance, a false alarm occurs on no-change trials when the working memory layer loses a peak for one of the items in the sample array, disinhibiting sites tuned to the item in the contrast layer. This leads the contrast layer to detect the item as novel. These errors are more common in children than adults.

Simulating the visual change detection task built upon the simulations of both spatial cognition and infant visual exploration. The task structure is similar to position discrimination, with the comparison of sequentially-presented stimuli used to generate a same/different judgment on each trial. Unlike position discrimination, however, the stimuli include multiple items at set sizes greater than one, similar to the infant change preference task. Johnson et al. (2014) situated the DNF model equipped with the same/different decision system in the change detection task to illustrate how this architecture could realize VWM capacity limits in adults. These simulations showed that the recurrent excitatory and inhibitory interactions within the contrast and working memory layers constrains the number of items that can be maintained simultaneously: more peaks in the working memory layer generate more inhibition, which feeds back into both the contrast layer and working memory layer to prevent more peaks from forming. Quantitative fits of adults’ performance in the change detection task indicated that the model had to be able to hold up to six items in memory at once to produce capacity estimates between four and five items, while also producing the same pattern of errors as adults across set sizes and trial types.

With the demonstration by Johnson et al. (2014) that the model could account for adults’ performance and capacity limits in the change detection task, Simmering (2016; see also Simmering et al., 2015) next asked whether the Spatial Precision Hypothesis could account for age-related changes in performance during childhood as well. Strengthening connectivity in the model over development had three consequences for change detection performance. First, it increased the stability of working memory peaks, which led them to be less susceptible to interference from noise and other items in working memory, meaning that once an item was encoded it was more likely to be maintained. Second, reduced interference allowed the model to maintain more items simultaneously throughout the delay, effectively increasing its capacity. Third, the model more robustly detected change. The result was more correct identification of same on no-change trials (correct rejections) and fewer misses on change trials (i.e., more hits), even if the number of items held in working memory did not change. Together the simulations by Johnson et al. and Simmering demonstrate that the same neurocognitive and behavioral processes applied across spatial cognition and infant visual exploration could also account for age-related change VWM capacity during childhood.

#### Reconciling Inconsistent Capacity Estimates Across Tasks and Development

The implementation of both the infant change preference and visual change detection tasks within a single model architecture allowed Simmering (2016) to address an inconsistency in the literature: behavioral results with infants suggested that capacity reached adult-like levels by 10 months of age (Ross-Sheehy et al., 2003), whereas a wide range of studies showed continuous increases in capacity during childhood as measured by visual change detection (e.g., Cowan et al., 2005; Riggs et al., 2006; Simmering, 2012). Previous simulations of infants’ and adults’ performance suggested one possible explanation for this discrepancy, as estimates from the change preference task were higher than the number of peaks held in the model (Perone et al., 2011) but estimates from the change detection task were lower than the number of peaks (Johnson et al., 2014). Simmering sought to test the model’s account of capacity limits across tasks, both empirically and computationally, by testing young children in both behavioral tasks and quantitatively fitting performance over development using the Spatial Precision Hypothesis.

Simmering (2016) generated three empirical predictions and one simulation prediction. First, she predicted that estimates of young children’s capacity in the change preference task would be higher than the three to four items estimated in infancy. Second, she predicted when the same participants were tested in both tasks, the change preference task would yield higher estimates than change detection. Third, despite these discrepant estimates, Simmering predicted performance would be correlated across tasks because both tasks depended on the same underlying neurocognitive processes of recognizing familiarity and detecting novelty. Lastly, the computational prediction Simmering tested was changes in connectivity derived from the Spatial Precision Hypothesis could account for developmental changes in performance across both tasks.

Simmering’s (2016) empirical results supported these predictions: all age groups showed estimates of at least six items (the highest set size tested) in change preference, but about two to four items over development in change detection. Despite these different estimates, children’s performance was positively correlated across tasks when comparing the respective behaviors reflecting recognition (switching between displays in change preference, correct rejections in change detection) and novelty detection (preference scores in change preference, hits in change detection) across tasks. Testing these specific correlations was motivated directly by the model’s account of behavior, rather than the prior literature in which the magnitude of a preference score is typically not interpreted meaningfully, and switches in looking tasks are attributed to attention rather than memory formation and recognition.

Lastly, Simmering’s (2016) simulations of development showed the patterns from early childhood to adulthood in each task could arise through the Spatial Precision Hypothesis. Furthermore, simulating both tasks in the same age group showed how the structure of these tasks affected the functioning of the memory system. Similar to prior simulations (Perone et al., 2011; Johnson et al., 2014), Simmering found different relations between the number of peaks held in memory and estimates of capacity from the model’s performance, with the change preference task over-estimating capacity and change detection under-estimating capacity. However, Simmering also showed that the same memory system could indeed hold more items in the more supportive change preference task through the repeated presentations (see Simmering, 2016, Chapter 4). These empirical and computational results addressed gaps in the VWM literature regarding how capacity estimates were understood across tasks, the source of age-related increases in capacity, and the reasons capacity is limited. These insights were gained by specifying how cognition and behavior relate within each task.

#### Synthesis of Visual Working Memory Capacity

Emergence is a central concept for understanding how capacity limits arise and influence behavior. The number of items that can be held in memory is an emergent produce of the nature of the interactions that encode, maintain, and compare representations. Even when items are held accurately in memory, the way in which those representations must be used (fixations in the change preference task versus same/different decisions in the change detection task) may over- or under-estimate the contents of memory. These processes are all affected by changes in stability, with increasing stability allowing for more items to be encoded and maintained as well as more accurate comparison and decision processes, and smaller differences across task contexts (Simmering, 2016). Simulating the performance in the change-preference task for infants and for young children and adults highlights the continuity of neurocognitive processes that support looking task behavior over development. Showing how the same model could predict connections in performance from this looking task to the canonical change detection task with young children and adults provides strong support for continuity of processes across tasks.

### Model Simulations Summary

The simulations we presented here illustrate the key theoretical constructs of emergence, stability, and continuity in behavior, cognition, and development. Table 1 summarizes the simulations we have described, focusing on the central changes in cognitive processes that arise through increased stability and the correspondence of these changes to behavioral phenomena. Increased stability over development has five important consequences illustrated in these simulations: (1) activation builds more quickly in the contrast layer for faster encoding; stronger activation produces peaks in the working memory layer that are (2) encoded more accurately and (3) maintained more accurately through delays (i.e., less likely to drift or “die out”); (4) increased accuracy of peaks, along with stronger associated inhibition, leads to more precise discrimination through a more robust comparison process; and (5) stronger activation and long-term memory traces support simultaneous encoding and maintenance of more items. Although, these changes all arise from implementing the Spatial Precision Hypothesis, we highlight only the most relevant changes in processes per task.

**Table 1:** Cognitive and behavioral consequences of increased stability over development.

Task	Cognitive processes	Behavioral phenomena
A-not-B	On the first B trial, representation of B more likely to be maintained through the delay	Less likely to show perseverative reaching on first B trial
Spatial recall	Stronger and more precise representations of midline and targets	Transition in bias relative to midline (from attraction to repulsion), decreased magnitude of errors, narrowing range of attraction toward previously-remembered locations
Position Discrimination	Stronger and more precise representations of midline and targets	Decreased JNDs, transition in effects of direction (better discrimination in opposite direction of drift seen in recall)
Habituation	Representations build more quickly and are maintained more accurately, with faster and more accurate comparison	Faster habituation, better discrimination of similar items
Visual paired comparison	Representations build more quickly and are maintained more accurately, with faster and more accurate comparison	Faster shift rate, shorter look durations, better discrimination of similar items
Infant change preference task	Representations build more quickly and are maintained more accurately, with faster and more accurate comparison	Robust change preference at higher set sizes, faster shift rate
Visual change detection	More representations maintained simultaneously, more accurate comparison and robust signals for response	Higher capacity estimates, fewer errors on no-change trials relative to change trials

Emergence is illustrated in Table 1 through the differences in which cognitive processes are most relevant to each task as well as the correspondence of the same cognitive process to different outcomes across tasks. For example, one consequence of increased stability is how quickly input is encoded; this change is most dramatic during infancy (Perone and Spencer, 2013b) but continues to occur through early childhood into adulthood (Simmering, 2016). However, this developmental change has little influence in the Piagetian A-not-B task or spatial recall because these tasks allow ample time for slower encoding. Across tasks that use shorter stimulus presentations, the effect of faster encoding on behavior differs according to how representations are used in the task. In looking tasks, faster encoding leads to faster recognition of familiarity and release of fixation, which corresponds to habituation rates or shift rates depending on whether the task includes one or two stimuli. In capacity-related tasks, faster encoding allows more items to be encoded, either across repetitions in the infant change preference task, or in a single presentation of the visual change detection task. By understanding how one cognitive system is coupled to behavioral systems, we gain a clearer picture of how a single developmental mechanism yields a vast array of performance differences across specific task contexts. The focus on behavior as an emergent process has also provided a clearer picture of continuity of cognitive and developmental processes within and across tasks. As Table 1 shows, developmental change in stability corresponds to multiple cognitive changes that influence behavior across tasks and domains.

Despite the successful applications of the DNF model, there are some notable shortcomings. One shortcoming is DNF models focus primarily on how activation changes in real time to produce behavior, with less emphasis on the processes that support learning and development (see Schlesinger and McMurray, 2012, for discussion of timescales in models). Specifically, the only learning implemented in these model simulations was a simple Hebbian mechanism that accumulates a history from above-threshold activation in the excitatory layers. Although this long-term memory trace was sufficient to capture key behavioral characteristics of the tasks we described here, it would likely not be capable of many forms of learning found in empirical studies. Developmental change in the simulations described here were implemented “by hand”, that is, by iterating through slightly stronger or weaker parameter values until the model produces a satisfactory fit to behavioral data, rather than simulating the developmental process itself (but see Perone and Spencer, 2013a). As reviewed by Schlesinger and McMurray (2012), the processes underlying learning and development have been a more central focus for connectionist and rational/symbolic (e.g., Bayesian, ACT-R) models.

Another shortcoming is that the model has not, and likely cannot, simulate all the variants of the tasks presented here. For example, in the visual change detection paradigm, objects were represented as a feature value along a single dimension (i.e., color) which makes it impossible to simulate more complex visual arrays. Furthermore, the model simulations we presented only include one set of neurocognitive processes representing visuospatial information. Although these processes are involved in a broad range of behaviors, they are clearly not the only neurocognitive architectures needed to adapt across contexts.

## Theoretical Implications and Concluding Remarks

Early psychological theorist envisioned the “grand theory” of fundamental principles that explain human and animal behavior (for discussion, see Gibson, 1994). The DNF model and DFT principles it is grounded in are anchored to this historical vision and aspire to identify a set of principles to describe a wide array of behaviors (Spencer et al., 2006). The study of cognition and its development has instead long been partitioned into sub-domains due to the complexity of the processes under investigation. A by-product of this partitioning is minitheories that may or may not be able to be combined into a larger whole. The simulations we reviewed show how one neurocognitive system connect phenomena using different behavioral tasks over development. The models were initially developed to explain behavior in their own domain and developmental periods, but our synthesis shows that connecting them into a bigger whole exemplifies how general theoretical constructs can explain behavior, cognition, and development.

Our synthesis of DNF model simulations has implications for long-standing debates in psychology. One debate is centered on whether perception and cognition are separable processes and, in particular, the influence of cognition on perception (for discussion, see Firestone and Scholl, 2016). In the DNF model, perceptual (encoding in the contrast layer) and cognitive (memory formation in the working memory layer) are interdependent. This interdependency is apparent in simulations of visual change detection. When the working memory layer loses a peak associated with one of the items on the sample array, it perceives that item as new when the test array appears, leading to a false alarm. Another long-standing debate is whether cognition is domain-specific (see Karmiloff-Smith, 1992, 1994, for review): the domain specific view posits that cognitive processes distinctly correspond to domains, whereas the domain general view is that a set of basic cognitive processes apply across domains. Our synthesis of DNF model simulations showed how the same neurocognitive processes can support different behaviors across a wide array of tasks and developmental periods. The specification of the basic neurocognitive processes of encoding, maintenance, comparison, and long-term memory formation with different behavioral system in the DNF model allowed these connections to be made concretely.

Our synthesis of DNF model simulations also has implications for our understanding of behavior. The first implication is there is not a one-to-one mapping between behavior and cognition; rather, behavior emerges in context. For example, tasks designed to assess visual working memory capacity assumed that the target behavior (i.e., longer looking to the changing display in the change preference task; hits and correct rejections in change detection) was evidence that displays’ contents were held in memory. However, our simulations showed that these tasks systematically over- or under-estimated the contents of memory due to the differential support needed for looking or same/different decisions, respectively. The DNF model uses whatever it has available (e.g., memory of two of four items in the task space) to behave. A classic example of emergence in a developing system is the stepping reflex (Thelen et al., 1984). Newborn infants show step-like alternating leg movements when held upright over a flat surface; around 4 months of age, this behavior disappears, but then reappears as infants begin to walk. The pattern was originally attributed to a decrease in reflexive movement (causing the disappearance of the behavior) followed by an increase in voluntary control of movement (causing the reappearance of the behavior). Thelen and colleagues proposed the u-shaped developmental pattern in behavior was not caused by an internal transition but rather the contribution of the pull of gravity on infants’ legs as they gained fat more quickly than muscle during early infancy. They showed that the stepping behavior was an emergent product of internal and external forces by submerging non-stepping infants’ legs in water – thus reducing the influence of gravity – which allowed the stepping behavior to occur. This example highlights the need to interpret behavior in context and not assign priority to internal components over external components (for further discussion, see Fogel and Thelen, 1987).

The second implication is cognition should be viewed as emerging in the context of the body perspective. For example, the act of looking structures memory formation which, in turn, contributes to the maintenance or release of fixation. There is a continuous, mutually influential loop between cognition and behavior (for similar discussion, see Pezzulo and Cisek, 2016). A striking empirical example of cognition being structured in relation to the body in early development is in the A-not-B task. In particular, when young infants who normally make the A-not-B error are stood up in between the A trials and B trials, they no longer make the error (Smith et al., 1999).

The third and perhaps most crucial implication of our synthesis is stability is a critical component of behavior in the moment as well as a domain-general developmental mechanism. Stability refers to how reliably a system can exhibit a given state; Stability is a more general concept and can be seen in the motor domain. A recent study by D’Souza et al. (2017) showed infants exhibit age-related decreases in the number of extraneous movements they make when executing an intentional action (e.g., moving their feet when reaching) over the second half of the first year. These observations can be understood as an age-related increase in stability which improves suppression of competition (e.g., other potential actions) and robust execution of an action (e.g., reaching to an object). This very process was observed the DNF model’s account of the A-not-B task. Stability enabled the model to form a stable working memory peak on the B trial, suppressing competition from prior reaches to A, and guiding a correct reach to B.

In closing, simulations of the DNF model support the notion transitions in cognitive development are not qualitative in nature (for discussion, see Kagan, 2008) but reflect the organization of a system in a task context with specific behavioral demands. Cognition and behavior emerge in context, and in order to understand how they change over development, we must also understand how they operate in real time in specific task contexts. We contend our synthesis offers general theoretical implications applicable beyond the realm of modeling. We can, as a field, take our theoretical understanding of performance in specific tasks and “put the pieces together again” (Oakes, 2009) to achieve our larger goal to understand the adaptability of human behavior. This, we believe, will bring us closer to the grand theory.

## Author Contributions

Both authors contributed equally to the development and writingof this manuscript.

### Conflict of Interest Statement

VS is now employed by the non-profit ACT, Inc.

The remaining author declares that the research was conducted in the absence of any commercial or ﬁnancial relationships that could be construed as a potential conﬂict of interest.
